# Oral immunization with *Shigella sonnei* WRSs2 and WRSs3 vaccine strains elicits systemic and mucosal antibodies with functional anti-microbial activity

**DOI:** 10.1128/msphere.00419-23

**Published:** 2023-12-22

**Authors:** Shikha Shrivastava, Alain B. Agnememel, Esther Ndungo, Dilara Islam, Yuanyuan Liang, Robert W. Frenck, Marcela F. Pasetti

**Affiliations:** 1 Department of Pediatrics, Center for Vaccine Development and Global Health, University of Maryland School of Medicine, Baltimore, Maryland, USA; 2 Department of Epidemiology and Public Health, University of Maryland School of Medicine, Baltimore, Maryland, USA; 3 Division of Infectious Diseases, Cincinnati Children’s Hospital Medical Center, Cincinnati, Ohio, USA; University of Florida, Gainesville, Florida, USA

**Keywords:** *Shigella*, oral vaccines, functional antibodies, mucosal immunity

## Abstract

*Shigella* causes bacillary dysentery and is responsible for a high burden of disease globally. Several studies have emphasized the value of functional antibody activity to understand *Shigella* immunity and correlates of protection. The anti-microbial function of local (mucosal) antibodies and their contribution to preventing *Shigella* infection remain unknown. The goal of this study was to identify the functional humoral immune effectors elicited by two *Shigella sonnei* live oral vaccine candidates, WRSs2 and WRSs3. Complement-dependent bactericidal [serum bactericidal antibody (SBA)/bactericidal antibody (BA)] and opsonophagocytic killing antibody (OPKA) activity were determined in sera and stool extracts as indicators of systemic and local anti-microbial immunity. High levels of SBA/BA and OPKA were detected in serum as well as in fecal extracts from volunteers who received a single dose of WRSs2 and WRSs3. Functional antibody activity peaked on days 10 and 14 post-vaccination in fecal and serum samples, respectively. Bactericidal and OPKA titers were closely associated. Peak fold rises in functional antibody titers in serum and fecal extracts were also associated. Antibody activity interrogated in IgG and IgA purified from stool fractions identified IgG as the primary driver of mucosal bactericidal and OPKA activity, with minimal functional activity of IgA alone, highlighting an underappreciated role for IgG in bacterial clearance in the mucosa. The combination of IgG and IgA in equal proportions enhanced bactericidal and OPKA titers hinting at a co-operative or synergistic action. Our findings provide insight into the functional anti-microbial capacity of vaccine-induced mucosal IgG and IgA and propose an operative local humoral effector of protective immunity.

**IMPORTANCE:**

There is an urgent need for a safe, effective, and affordable vaccine against *Shigella*. Understanding the immunological underpinning of *Shigella* infection and the make-up of protective immunity is critical to achieve the best approach to prevent illness caused by this mucosal pathogen. We measured the complement-dependent bactericidal and opsonophagocytic antibody killing in serum and stool extracts from adult volunteers vaccinated with *Shigella sonnei* live oral vaccine candidates WRSs2 and WRSs3. For the first time, we detected functional antibody responses in stool samples that were correlated with those in sera. Using purified stool IgA and IgG fractions, we found that functional activity was mediated by IgG, with some help from IgA. These findings provide insight into the functional anti-microbial capacity of vaccine-induced mucosal IgG and IgA and support future studies to identify potential markers of protective mucosal immunity.

## INTRODUCTION

*Shigella* is a food- and water-borne pathogen that causes acute, inflammatory diarrhea. It is responsible for high burden of disease globally with an attributable death rate of 148,000 annually (1). The most affected are children under 5 years of age living in poor resource areas, with those between 2 and 3 years of age having the highest incidence of disease (2). Untreated *Shigella* infection during childhood has been associated with linear growth faltering in infants and toddlers (3), which in turn leads to poor health and impaired development (4). In addition, *Shigella* has become a major public health concern due to the rapid rise in resistance to conventional anti-microbial treatment (ciprofloxacin or azithromycin) (5–8), which is compounded by the bacteria’s ease of transmission and infectivity (9).

Four species of *Shigella* have been identified based on serological typing: *Shigella flexneri*, which has 15 serotypes and is the most prevalent in low-income countries; *Shigella sonnei,* which has only one serotype and found mainly in industrialized regions; *Shigella dysenteriae*, which has historically caused epidemic outbreaks of high case fatality; and *Shigella boydii*, which has 19 serotypes and is associated with a small number of cases, mainly in South Asia. A substantial change in the distribution of *Shigella* serotypes has been seen in recent decades with a marked increase in the global burden of *S. sonnei* (10–12). Compared to *S. flexneri, S. sonnei* has shown greater capacity to acquire elements that confer antibiotic resistance (11), and this competitive advantage is surmised as a major driver of its expansion. There is therefore an urgent need for a safe, effective, and affordable vaccine against this preeminent gastrointestinal mucosal pathogen, as none is available.

Epidemiological evidence from endemic areas indicates that while young children are at high risk for *Shigella* infection, the adult population is more refractory. This has been attributed to life-long exposure to *Shigella* eliciting natural immunity which, although imperfect, prevents re-infection (13–15). Hence, the existence of natural protective immunity acquired through natural contact with living organisms supports the feasibility of a live oral vaccine. Understanding the immunological underpinning of *Shigella* infection and the make-up of protective immunity is critical to achieve the best mucosal vaccine approach.

Historically, levels of serum antibodies against *Shigella* LPS have been associated with reduced incidence of shigellosis (16, 17). Several studies have emphasized the relevance of functional antibody activity to appraise *Shigella* immunity (18–21). Functional assays surpass measures of antibody binding in their categorical quantification of anti-microbial activity, which likely reflects an element of protective immunity. Complement-mediated bactericidal antibody (BA) activity has been detected in circulation following natural infection in adults (22) and children (23) living in areas where *Shigella* is endemic. Serum bactericidal antibody (SBA) titers have been reported to be inversely correlated with incidence of disease in controlled human infection models involving *S. flexneri* 2a (24) or *S. sonnei* (25). Increases in SBA titers have been reported in clinical studies that evaluated various vaccine candidates using different routes of immunization (26–30). Recent vaccine efficacy studies (Flexyn2a bioconjugate and the *S. sonnei* GMMA vaccine candidates) reported higher SBA titers in vaccine recipients who did not develop shigellosis after experimental challenge as compared to those with shigellosis (31, 32), as well as a linear relationship between vaccine efficacy and SBA titers at the time of challenge (31).

With the overarching goal of identifying functional humoral immune effectors as predictors of protective immunity and vaccine efficacy, we examined the anti-microbial activity of antibodies elicited by two live oral *S. sonnei* vaccine candidates, WRSs2 and WRSs3, administered to adult volunteers in a phase 1 clinical trial conducted at Cincinnati Children’s Hospital Medical Center (33). WRSs2 and WRSs3 are derivatives of the *S. sonnei* Moseley strain that are similar to the first-generation vaccine candidate, WRSS1, in their deletion of virulence plasmid-encoded VirG (IcsA) (34). Further attenuations, including deletion of the enterotoxin and acyl transferase genes, were introduced to increase tolerability, resulting in strains WRSs2 and WRSs3 (35, 36).

In the study presented here, we examined complement-dependent bactericidal and opsonophagocytic antibody killing activity in adult volunteers immunized with WRSs2 and WRSs3. Functional antibody responses were quantitated in sera as an indicator of systemic immunity and in stool samples as an indicator of local mucosal immunity. The contribution of each antibody isotype in anti-microbial function was interrogated in purified stool IgA and IgG fractions.

## RESULTS

### WRSs2 and WRSs3 elicited *S. sonnei-*specific systemic functional antibody responses

**Fig 1:**
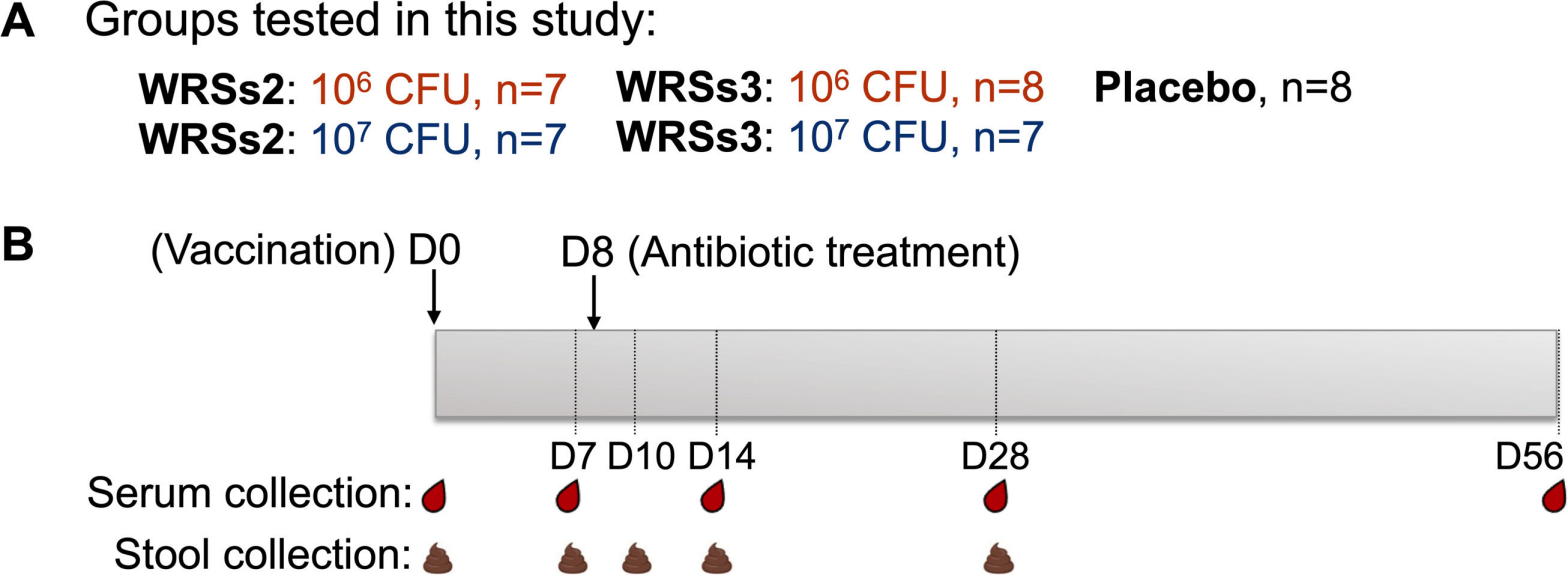
Schematic representation of groups and sample time points tested in this study. (**A**) Groups (WRSs2 and WRSs3 and placebo recipients) and dosage levels (10^6^ and 10^7^ CFU). (**B**) Individuals were vaccinated on day 0 (D0); all received antibiotic treatment on day 8. Serum was collected on days 0, 7, 14, 28, and 56. Stool was collected on days 0, 7, 10, 14, and 28.

To investigate the capacity of WRSs2 and WRSs3 vaccine candidates to elicit *S. sonnei*-specific functional antibodies, SBA and opsonophagocytic killing antibody (OPKA) activities were measured in a subset of serum samples from volunteers who participated in a dose-escalating phase I study that assessed the safety and immunogenicity of the live attenuated *S. sonnei* vaccines WRSs2 and WRSs3 (33). In our study, we tested samples from a total of 37 adult individuals who received the two highest doses, 10^6^ colony forming units (CFU) or 10^7^ CFU, of either WRSs2 (*n* = 14), WRSs3 (*n* = 15), or placebo (*n* = 8). A total of 166 serum samples were available for testing. A schematic representation of volunteer numbers per group and time points post-vaccination is shown in Fig. 1. The vaccine was administered as a single oral dose on day 0. Sera were obtained on days 0, 7, 14, 28, and 56 after vaccination. Demographic characteristics of the enrolled individuals as well as vaccine tolerability have been described in detail elsewhere (33). Vaccine responses, including LPS-specific IgG and IgA measured in serum, fecal supernatants, and antibody in lymphocyte supernatants (ALS), have been reported previously (33, 37).

**Fig 2:**
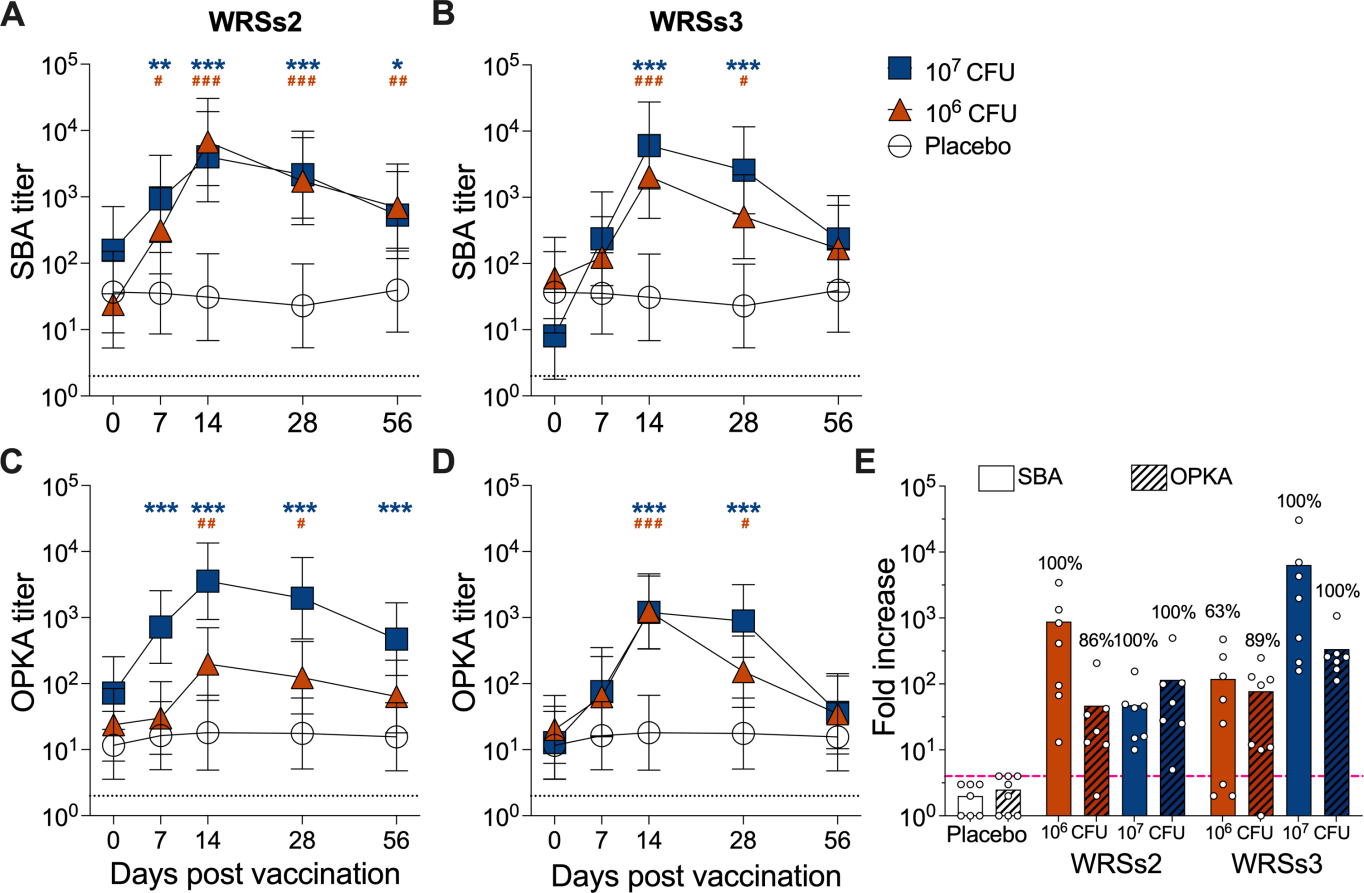
Kinetics of functional antibody responses in circulation in volunteers orally immunized with *Shigella* WRSs2 or WRSs3 vaccine strains. SBA and OPKA titers before and after oral immunization with WRSs2 (**A, C**), WRSs3 (**B, D**), or placebo. Symbols represent log_10_ geometric mean titers ± 95% CI. Clear circles represent the placebo group, orange triangles represent individuals vaccinated with the 10^6^ CFU dose, and blue squares represent individuals vaccinated with the 10^7^ CFU dose. Blue asterisks and orange pound signs indicate statistically significant differences for each vaccine/dose response at defined time points as compared to placebo. * and #, *P* < 0.05; ** and ##, *P* < 0.01; *** and ###, *P* < 0.001 determined by linear mixed effect model after log transformation of individual endpoint titers. Dotted line indicates limit of detection (titer of 2 for SBA and OPKA). (**E**) Largest fold increase in titers over baseline at any day post-vaccination within the treatment groups. Symbols represent individual fold increase in titers. Numbers above bars represent percent of subjects that had ≥4-fold increase (pink dashed line) in antibody titers compared to baseline titers at any time point after vaccination.

SBA and OPKA responses following WRSs2 and WRSs3 vaccination are summarized in Fig. 2; Table S1. Volunteers who received either vaccine, regardless of the dosage level (10^6^ CFU or 10^7^ CFU), developed strong SBA and OPKA responses. Functional antibody activity was detectable at the earliest time point (day 7) post-vaccination in almost all vaccine groups. Mean SBA and OPKA titers peaked on day 14 and remained above baseline on day 28 (Fig. 2; Table S1). Functional antibody titers dropped by day 56 but were still differentiable from baseline for the 10^6^ CFU dose of WRSs2 (SBA), 10^7^ CFU dose of WRSs2 (OPKA), and 10^7^ CFU dose of WRSs3 (SBA) (Table S1). Of note, these same groups also had significantly higher functional antibody titers compared to baseline on day 7 (Table S1).

A similar trend was observed when SBA and OPKA responses were compared between vaccinated and placebo groups (Fig. 2; Table S1). Volunteers who received either 10^6^ CFU or 10^7^ CFU of WRSs2 had higher SBA titers than those in the placebo group at all time points post-vaccination (days 7, 14, 28, and 56, Fig. 2A). Similarly, OPKA titers were significantly higher on all days for the 10^7^ CFU dose group, but only on days 14 and 28 for the 10^6^ CFU dose group (Fig. 2C). All volunteers who received the WRSs3 vaccine had significantly higher SBA and OPKA titers on days 14 and 28, regardless of the dose. Titers had dropped by day 56 to levels no different from those of the placebo (Fig. 2B and D).

Post-vaccination SBA and OPKA titers were also compared to titers measured before vaccination (baseline) and maximum fold increases determined (Fig. 2E). All vaccines and both dosage levels resulted in ≥45-fold increase in mean titers compared to baseline. Mean fold increases were highest for the 10^7^ CFU dose of WRSs3: 6,384-fold for SBA and 339-fold for OPKA. Rates of seroconversion were defined as the proportion of individuals exhibiting greater than a fourfold increase in SBA or OPKA titer above baseline at any time point post-vaccination (dotted line in Fig. 2E). All individuals immunized with WRSs2, regardless of the dosage level received, seroconverted for SBA. The same was observed for those who received the 10^7^ CFU dose of WRSs3, whereas 63% seroconversion was observed among those who received the lower dose, 10^6^ CFU (Fig. 2E). When considering OPKA responses, >85% of vaccinated individuals seroconverted regardless of the vaccine or dosage level received (Fig. 2E). One hundred percent functional seroconversion was achieved by both vaccines given at the highest dose. No seroconversion was observed in individuals in the placebo group.

### WRSs2 and WRSs3 induced *S. sonnei*-specific mucosal functional antibody responses

**Fig 3:**
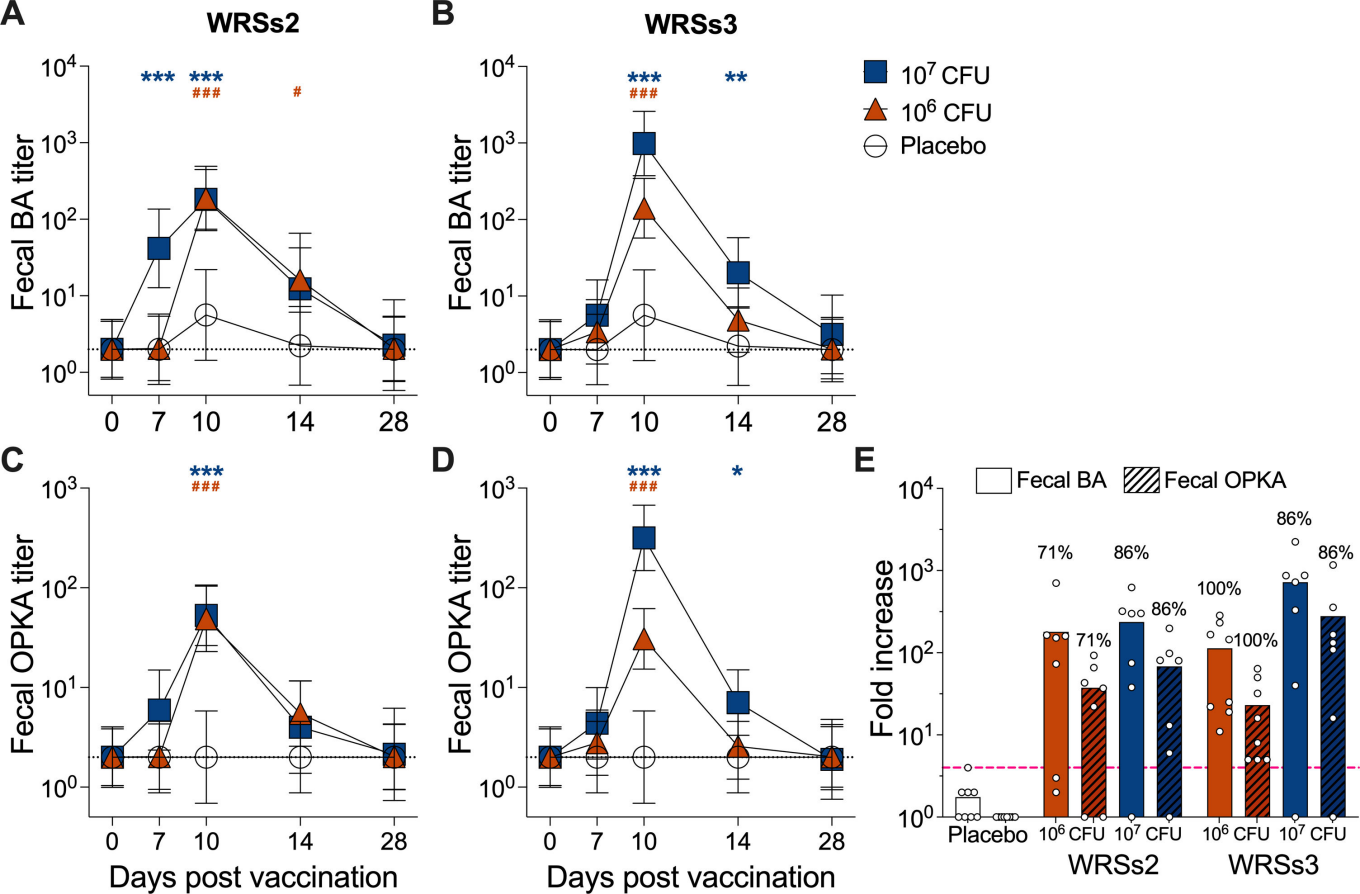
Kinetics of the mucosal (fecal) functional antibody response after vaccination. Fecal BA and OPKA titers before and after administration of WRSs2 (**A, C**) or WRSs3 (**B, D**) vaccine or placebo. Symbols represent log_10_ geometric mean titers ± 95% CI. Clear circles represent the placebo group, orange triangles represent individuals vaccinated with the 10^6^ CFU dose, and blue squares represent individuals vaccinated with the 10^7^ CFU dose. Orange and blue asterisks indicate significantly higher titers, compared to placebo, in individuals vaccinated with the 10^6^ CFU or 10^7^ CFU dose, respectively. * and #, *P* < 0.05; ** and ##, *P* < 0.01; *** and ###, *P* < 0.001 as determined by linear mixed effect model after log transformation of individual endpoint titers. Dotted line indicates limit of detection (titer of 2 for BA and OPKA). (**E**) Largest fold increase in titers over baseline at any day post-vaccination within the treatment groups. Symbols represent individual fold increase in titers. Numbers above bars represent percent of subjects that had ≥4-fold increase (pink dashed line) in antibody titers compared to baseline titers at any time point after vaccination.

We next evaluated the presence of *S. sonnei*-specific functional antibody activity locally. The functional antibody assays applied to sera were modified to determine BA and OPKA activity in fecal extracts. Time points investigated included days 0, 7, 10, 14, and 28 after vaccination (Fig. 1). The same volunteers included in the serum antibody analysis were examined for fecal functional antibodies (Fig. 3 ; Table S2). A total of 141 stool samples were available for testing.

All study participants were treated with ciprofloxacin starting on day 8 post-vaccination. To ensure that the only bactericidal activity measured was strictly antibody-mediated and not non-specific killing due to the presence of antibiotics, all fecal supernatants were buffer-exchanged into 1× phosphate-buffered saline (PBS; pH 7.4) using a centrifugal concentrator with a molecular weight cutoff (MWCO) of 10,000 Da following the stool processing. The detailed preparation of the fecal supernatant for the stool functional antibodies testing is described in the Materials and Methods section.

Fecal bactericidal and OPKA responses are shown in Fig. 3. Both vaccines at the two dosage levels tested attained peak BA and OPKA responses 10 days post-vaccination, and these responses were significantly higher compared to baseline (Table S2). BA and OPKA titers remained elevated through day 14 in the high dose WRSs3 recipients and in individuals vaccinated with 10^6^ CFU dose of WRSs2 (Table S2). By day 28, fecal BA and OPKA titers had returned to baseline levels in all groups.

The same trend was observed when fecal BA and OPKA responses in the vaccinated groups were compared to placebo recipients (Fig. 3; Table S2). Fecal BA and OPKA titers in vaccinated individuals, regardless of vaccine or dose group, were significantly higher than those of the placebo group on day 10 post-vaccination (Fig. 3A through D). Fecal BA titers were already elevated on day 7 post-vaccination in volunteers who received WRSs2 vaccine at the 10^7^ CFU dose (Fig. 3A). In the WRSs3 recipients, regardless of the dose, fecal BA and OPKA were also significantly elevated on day 14 as compared to placebo (Fig. 3B and D).

Mean fold increases for all vaccines and dosage levels were at >20-fold compared to baseline (Fig. 3E). As was observed in serum, the 10^7^ CFU dose of WRSs3 resulted in the highest mean fold increases: 723-fold for fecal BA and 279-fold for fecal OPKA. Seroconversion rates calculated for fecal BA and OPKA were similar for both WRSs2 and WRSs3 (Fig. 3E). Among those vaccinated with the 10^6^ CFU dose of WRSs2, 71% seroconverted, while 86% vaccinated with the 10^7^ CFU dose of WRSs2 seroconverted. All subjects vaccinated with 10^6^ CFU of the WRSs3 vaccine seroconverted, compared to 86% of those vaccinated with the higher dose. Eighty-six percent functional seroconversion was achieved by both vaccines given at the highest dose.

### Systemic and mucosal functional antibody responses were correlated

**Fig 4:**
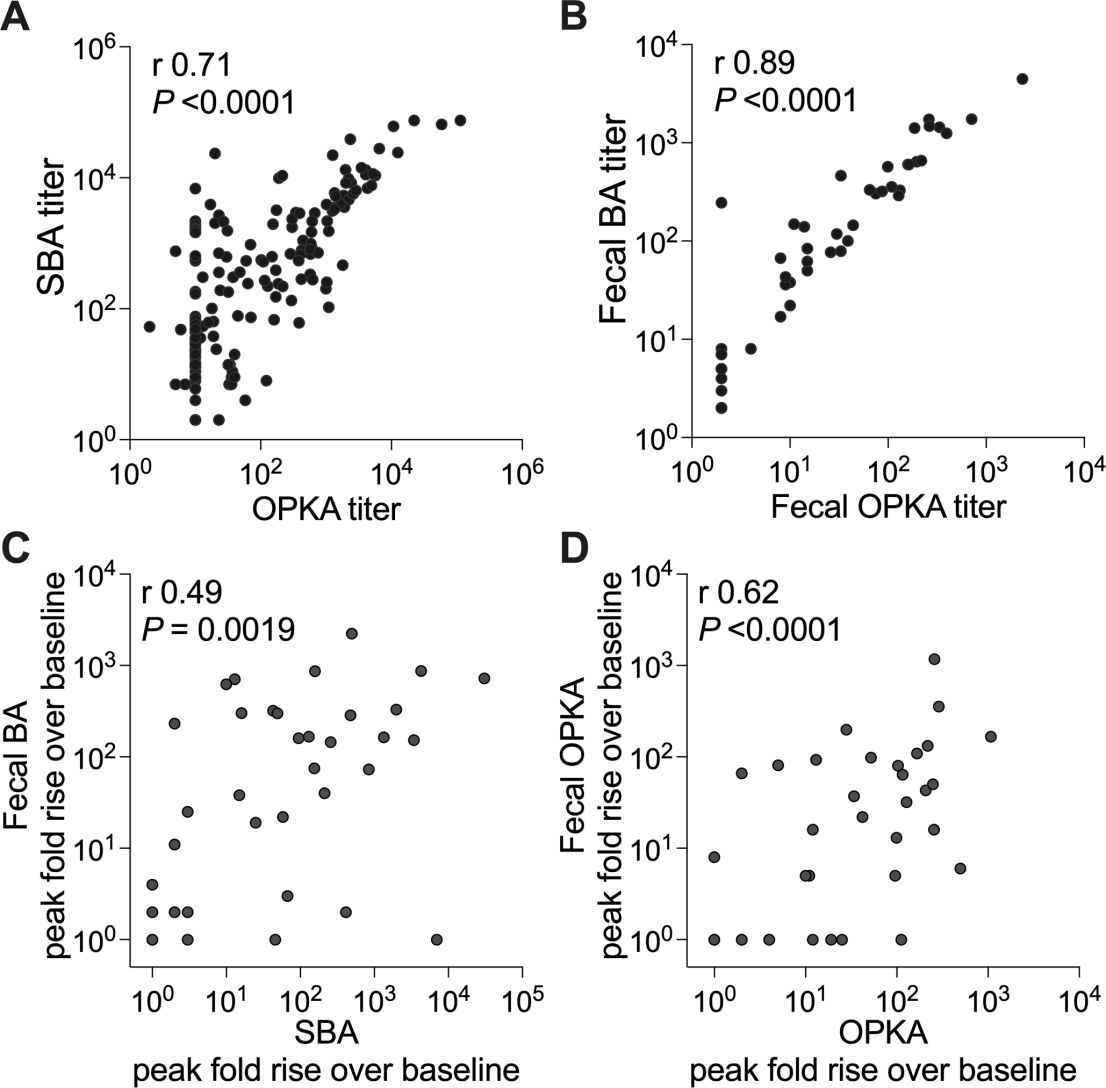
Correlations between bactericidal antibody responses. (**A**) Correlation between serum BA and OPKA titers and (**B**) between fecal BA and fecal OPKA titers; data represent titers from all 37 individuals (both vaccinated and placebo groups) and available time points tested (see Fig. 1). *n* = 166 for serum samples, *n* = 141 for fecal samples. (**C**) Correlation between the peak fold rise in serum BA and fecal BA titers (highest at any time post-vaccination over baseline) and (**D**) between serum OPKA and in fecal OPKA titers; data represent all 37 individuals tested (vaccinated and placebo groups, *n* = 37). Spearman’s *r* and *P*-values are shown within each graph.

SBA and OPKA titers were strongly associated (Spearman’s *r* = 0.71) (Fig. 4A). A similar correlation was found between functional antibody (BA and OPKA) titers in fecal extracts (Spearman’s *r* = 0.89, Fig. 4B).

We next investigated whether the functional antibody responses in systemic (serum) and mucosal (fecal extracts) compartments were associated, and for this analysis, we compared peak fold rises (highest fold rise over baseline at any time post-vaccination) in serum with peak fold rises in fecal antibody titers. A moderate positive correlation was observed between fecal and serum functional antibody levels (Spearman’s *r* = 0.49 and 0.62; Fig. 4C and D).

### Association between functional antibody activity and vaccine shedding

We also examined whether vaccine shedding was associated with functional antibody responses. Shedding was defined as the peak (maximum) vaccine shed by each participant (measured in CFU/g of stool) and the duration (measured in number of days) the vaccine was shed. These two outcomes were compared to the peak bactericidal and opsonophagocytic titers (highest at any time post-vaccination) and to the peak fold rise in functional antibody titers from each individual using Spearman’s correlation (Table S3).

In general, vaccine recipients with no or low shedding (i.e., <100 CFU, <1 day) had lower functional antibody levels as compared to subjects with more pronounced shedding (data not shown), albeit without a discernable trend. A strong positive correlation was observed between peak SBA fold increases and the magnitude of vaccine shed (Spearman’s *r* = 0.61) and the number of days the vaccine was shed (Spearman’s *r* = 0.61); all other associations with systemic or mucosal immune responses were modest (Table S3). When considering individuals in the WRSs2 or WRSs3 vaccinated groups separately, we found low or no association between shedding and functional antibody responses.

### Fecal IgG and IgA cooperation in functional bactericidal and opsonophagocytic killing activity

**Fig 5:**
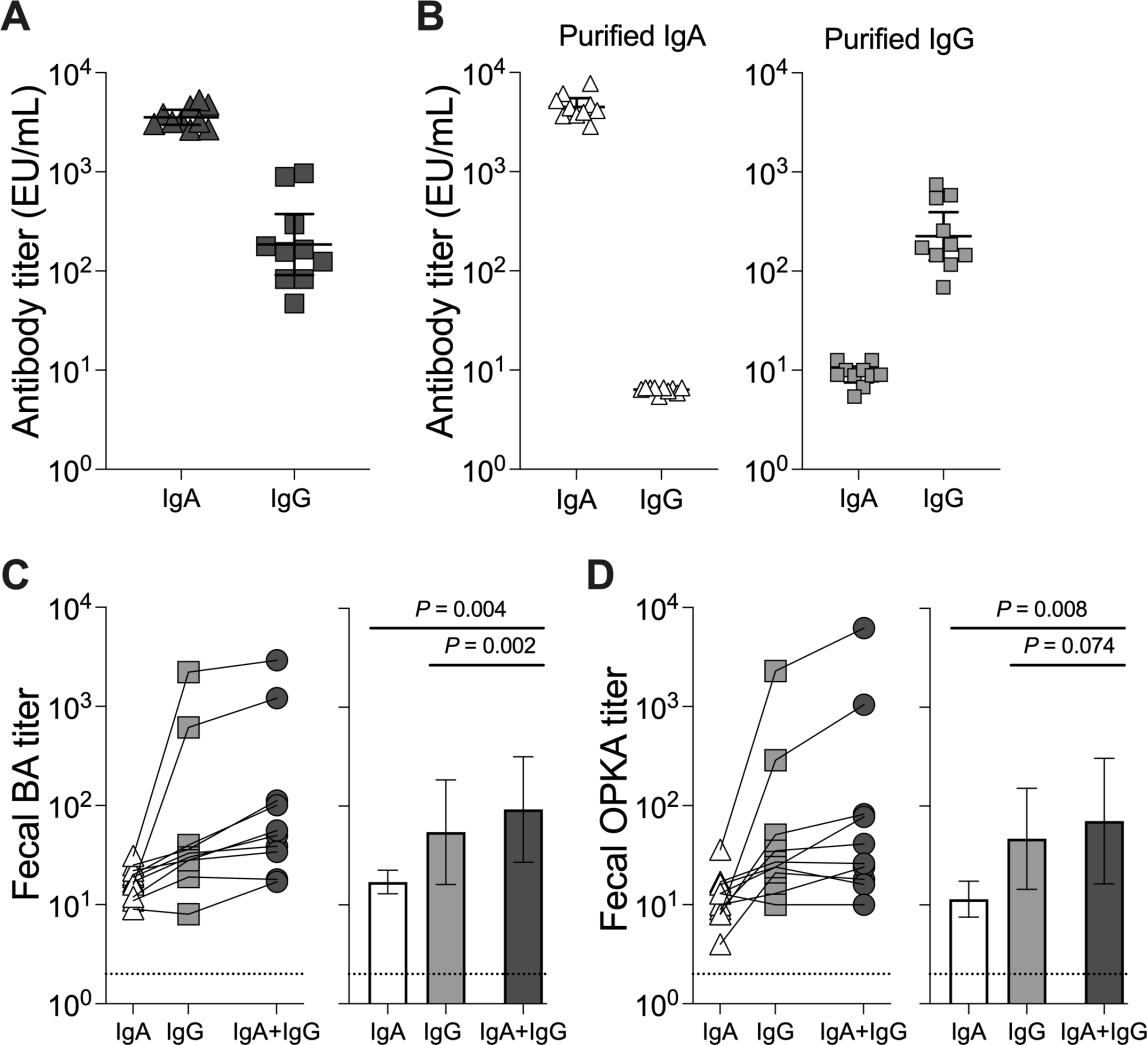
*Shigella* LPS-specific IgG and IgA contribute synergistically to bactericidal activity. (**A**) *S. sonnei* LPS-specific antibody isotypes in fecal supernatants from individuals at day 10 post-vaccination with 10^7^ CFU of either WRSs2 or WRSs3 (five individuals from each group). (**B**) *S. sonnei* LPS-specific antibody isotype in purified IgA and IgG preparations obtained from samples shown in panel **A**. (**C**) Fecal BA and (**D**) fecal OPKA titers of IgA, IgG, or a combination of IgA + IgG. Data represent individual samples (left) and means ± 95% CI (right). *P*-values obtained using Wilcoxon matched-pairs signed rank test are indicated in the graphs on the right. Dotted line indicates limit of detection (titer of 2 for BA and OPKA).

We have previously identified LPS IgG antibodies as drivers of serum bactericidal activity (38). To investigate the type of antibody involved in mucosal functional antibody activity, we selected day 10 fecal supernatants that exhibited high BA titers from individuals vaccinated with the 10^7^ CFU dose of WRSs2 or WRSs3 (five individuals from each group). The presence of *S. sonnei* LPS IgG and IgA antibodies in these supernatants was confirmed (Fig. 5A). IgG and IgA were purified from stool supernatants and preparations containing exclusively IgA or IgG were produced for each individual participant; the absence of other (depleted) antibody isotypes was confirmed by enzyme-linked immunosorbent assay (ELISA) (Fig. 5B). BA and OPKA assays were then performed in the purified IgA or IgG fractions as well as in preparations that contained a 1:1 mix of purified IgA and IgG (IgA + IgG) from each individual. To ensure equimolar antibody isotype concentrations in the IgA or IgG only versus the IgA + IgG mix, samples containing one isotype were prediluted 1:1 in PBS before being applied to the functional assays. All samples were tested at the same starting dilution.

BA and OPKA titers in the IgA-containing fraction were low (mean titers = 18.2 for BA and 13.4 for OPKA), but higher than the assay’s limit of quantification (dotted line in Fig. 5C and D), whereas high BA (mean titer = 305.5) and OPKA titers (mean titer = 277.9) were detected in the purified fecal IgG. Interestingly, fecal BA titers in the preparation containing both isotypes (IgA + IgG, mean titer = 456.4; Fig. 5C) were significantly higher than those detected in those containing IgA or IgG alone. The same was observed for fecal OPKA, except that the superior activity in the combined IgA + IgG preparation (mean titer = 754.7; Fig. 5D) vs IgG alone did not reach statistical significance (*P* = 0.074). In the majority of the samples tested (6/10), the bactericidal activity in the combined IgA + IgG preparations was greater than the sum of activity of each antibody isotype alone. OPKA activity that was greater in the combined IgA + IgG preparation than the sum of individual isotypes was also observed in 4 of the 10 samples tested. These findings suggest a synergy between the two antibody classes that enhances anti-microbial function.

## DISCUSSION

Vaccines against *S. sonnei* and/or other *Shigella* strains are in various stages of development (39). Oral delivery of live attenuated organisms is expected to mimic infection and activate mucosal immunity that would be evoked upon subsequent exposure. Robust, anamnestic, adaptive immunity within the local gut mucosa is critical to block *Shigella* at the site of entry.

In this study, we interrogated the capacity of WRSs2 and WRSs3—two representative clinically advanced oral vaccine candidates—to elicit functional antibodies that could engage in anti-microbial immunity *in vitro*. To this end, we determined the kinetics of antibody-mediated *S. sonnei* bactericidal and opsonophagocytic killing activity in serum and fecal extracts. Previous reports of WRSs2 and WRSs3 immune responses post-vaccination included *S. sonnei* LPS-, Invaplex-, and IpaB-specific antibodies in serum and ALS, antibody secreting cells, and stool IgA (33, 37).

We found that a single oral dose of WRSs2 or WRSs3 elicited robust SBA and OPKA titers in serum and fecal supernatants. The functional antibody responses measured in serum reached maximum levels on days 14 and 28 post-vaccination and persisted for at least 2 months (Fig. 2). The timing of SBA and OPKA responses is congruent with the kinetics of serum LPS IgG and IgA titers reported previously for individuals who participated in the same clinical study (33). The temporal overlapping of LPS-specific antibodies and SBA/OPKA titers is consistent with LPS being the main target of these anti-microbial functions.

Importantly, antibodies with bactericidal and opsonophagocytic killing activity were found in fecal supernatants as early as 7 days after vaccination, attaining a peak at day 10 post-vaccination. The peak time point for fecal bactericidal and OPKA responses observed in our study concurred with the time LPS IgA was found in stool and ALS in the same WRsS2 and WRsS3 vaccine recipients (33, 37). ALS have historically been considered a surrogate mucosal response as they reflect a humoral response elicited by mucosally primed gut-homing plasmablasts transiently in circulation while enroute to the lymph nodes or distant mucosal sites (37, 40, 41). The earlier peak response of fecal vs serum antibodies (10 days vs 14 days post-dosing, respectively) suggests a rapid anti-microbial response in the colonic mucosa following exposure. Fecal antibody activity quickly returned to baseline levels (by day 28), whereas serum antibody activity remained elevated up to 56 days post-vaccination in some groups. Interestingly, the groups that responded robustly early on (day 7) were those with longer-lasting immunity, suggesting that immune memory is a function of strength of priming. No overt differences were observed in the ability of WRSs2 and WRSs3 to elicit functional humoral immunity and the magnitude of responses; the vast majority of vaccinated subjects responded with functional antibodies (fourfold increase in titers over baseline) in serum and in stool. *Shigella* functional antibodies have been reported in serum from adult human volunteers following both parenteral and oral vaccination (26–30). To our knowledge, this is the first report describing the functional antibodies in mucosal samples and from individuals immunized with a *Shigella* vaccine candidate.

As *Shigella* does not cause invasive (bloodstream) infections, it has been puzzling to understand the contribution of antibodies with bactericidal and opsonophagocytic activity that are measured in serum in clearing this predominantly mucosal pathogen. The association of SBA (and OPKA) with clinical protection has been reported in several clinical studies; however, the extent to which these antibodies contribute to protection and their precise mode of action, including the location where they deploy these functions, is unclear. It is worth noting that vibriocidal (functional) antibody activity against another enteric pathogen, *Vibrio cholerae,* is known to correlate with clinical protection and is used as a surrogate of live cholera vaccine efficacy (42). Similar to what was observed for *Shigella* SBA and OPKA, vibriocidal antibodies are directed against *V. cholerae* LPS (43). It is reasonable to assume that there is redundancy in host responses to enteric pathogens, and a similar mechanism of functional antibody-mediated killing is deployed against both *V. cholerae* and *Shigella*.

The finding of antibodies in feces that can activate complement and kill *Shigella,* or facilitate uptake and killing of the organism by neutrophils, reveals that mucosal (local) functional antibodies are likely prominent mediators of protective immunity. Moreover, the correlation between functional antibody vaccine responses in feces and in serum suggests that vaccine-specific antibodies in circulation somehow reflect the humoral immune responses at the mucosal surface.

An interrogation of function in IgG and IgA purified from vaccine recipients’ stool samples revealed IgG as the main driver of mucosal bactericidal and opsonophagocytic killing activity, while activity attributable to IgA was minimal (20 times lower). Intriguingly, both bactericidal and opsonophagocytic killing activities were increased when IgG and IgA were combined, suggesting cooperation or synergy between the two antibody isotypes (Fig. 5). IgG has a strong affinity for complement proteins and activates the complement cascade via the classical pathway. IgA can activate complement via the alternative pathway (44–46). The enhanced bactericidal and opsonophagocytic activity when both IgG and IgA are present may reflect their combined contribution to each function, although this scenario would not explain the magnitude of such increase observed. Alternatively, it may reflect a more complex synergy whereby IgA, beyond deploying complement or phagocytic function, may be favoring clumping or agglutination (47, 48) that would facilitate IgG-efficient bactericidal and opsonophagocytic activity. Protective immunity in the gut is generally attributed to secretory IgA due to its abundance in mucosal surfaces (49). Here, our results implicate IgG as an important and underappreciated effector that can kill and block enteropathogens in the gut mucosa. Rather than either-or, a more efficacious anti-microbial effect is achieved through their combined action.

Even though IgG levels in the fecal supernatants were generally low (Fig. 5A), IgG’s robust antibacterial effect can be traced to its high binding capacity (avidity) to cognate antigen, complement activation, strong Fc interaction, and neutrophil engagement (OPKA). The anti-microbial properties of IgG within the gut mucosa observed here are in line with the high (>80%) protective efficacy of orally administered IgG-rich bovine colostrum with high anti-*S. flexneri* 2a LPS titers against infection with the same strain in humans (50). In fact, hyperimmune bovine milk has been used successfully as a prophylactic against diarrhea and illness caused by multiple enteric pathogens [reviewed in reference (51)]. Consistent with the presence of functional antibodies in feces (as mucosal secretions), we have detected LPS-specific IgG and IgA, and robust bactericidal activity in breast milk from mothers living in *Shigella*-endemic regions (data not shown). The anti-microbial activity of breast milk antibodies is expected to prevent *Shigella* infection in breastfed children.

Bactericidal and opsonophagocytic killing activity deployed by mucosal IgG and IgA *in vivo* (the scenario portrayed in our *in vitro* assays) would require the presence of complement within the gut. Complement is traditionally thought to be a systemic host defense system, with complement proteins produced in the liver and acting in blood and interstitial fluids (52). However, complement is also synthesized by cells in the human gut. In a proteomic analysis of human enteroids (primary epithelial cell monolayers), we identified the secretion of complement proteins C3 and C4 by the ileal epithelium; expression of these proteins was upregulated by human breast milk (53). Complement proteins have been detected in the gut lumen (54), and it has been suggested that they are activated and regulated by infiltrating cells; properdin, for example, is an alternative pathway complement regulatory protein produced by activated human neutrophils and secreted upon inflammatory cytokine signaling (55, 56). A recent study from Dennis Kasper lab showed that complement C3 was synthesized by three key types of cells in a microbiome-dependent manner and secreted into the intestinal lumen; the group reported luminal C3 to be critical for protection against enteric pathogens in mice infected with either *Citrobacter rodentium* or enterohemorrhagic *Escherichia coli* (57).

Shedding has been linked to immunogenicity of live oral *Shigella* vaccines (58, 59). We found that vaccine shedding was strongly positively correlated with serum bactericidal responses but not with mucosal functional antibodies. Venkatesan et al. reported an agreement between shedding and frequency of IgA ALS responders among WRSs3 recipients but no other clear associations (37). A more precise analysis of vaccine shedding (e.g., culture vs qPCR and time course) will help address its impact on local functional immunity in future studies.

The precise host factors and immune elements that help prevent *Shigella* infection in the human gut and their mode of action remain to be defined. The results described demonstrate for the first time the presence of *Shigella* IgG and IgA with effector functions against the intact organism (complement-mediated killing and opsonophagocytic killing) in the human gut. These antibodies were produced in response to oral vaccination with live attenuated strains; serum and fecal antibody responses were associated. Our results also shed light on the lead role of IgG and the synergistic action of IgG and IgA in anti-microbial activity *in vitro*. Further studies using purified antibodies (with defined specificity and representing different isotypes) and innate immune cells, and employing novel technologies such as the human enteroid model (60) and relevant animal models, are necessary to dissect the mechanisms by which mucosal antibodies and complement cooperate to clear enteropathogens in the gut.

## MATERIALS AND METHODS

### Clinical samples

The serum and fecal samples tested in this study were obtained in a phase I clinical trial of the WRSs2 and WRSs3 vaccine (NCT01336699). The study design, methods, vaccine safety, and immunogenicity endpoints were described elsewhere (33). The specimens tested in this study corresponded to volunteers who received the highest dosage levels (10^6^ and 10^7^ CFU) of WRSs2, WRSs3, or placebo; time points are shown in Fig. 1.

### Fecal supernatants

Frozen stool samples were thawed on ice, and a 200 mg aliquot of each stool sample was placed in tubes containing 500 mg of silica beads (BioSpec Products Inc, Bartlesville, OK) and 1 mL of extraction buffer containing soybean trypsin inhibitor-EDTA solution and phenylmethanesulfonyl fluoride solution (all from MilliporeSigma, St. Louis, MO). Stool samples were subjected to a 1-min bead beating cycle in a Bead Mill Homogenizer (VWR, Radnor, PA) followed by centrifugation at 14,000 rpm for 30 min at 4°C. The resulting supernatants were harvested and filtered through a 0.22 µm syringe filter (MilliporeSigma). An additional buffer-exchange step was included for fecal supernatants harvested from day 10 and day 14 samples to remove traces of antibiotic that would interfere in the functional activity readout. Equal volumes of fecal supernatant were buffer exchanged into 1× PBS, pH 7.4 (Thermo Fisher Scientific, Waltham, MA) by centrifuging at 4,000 rpm in a Vivaspin 20 (Sartorius, Bohemia, NY) centrifugal concentrator (MWCO 10,000 Da); the procedure was repeated three times with fresh PBS. The filtered fecal supernatants were stored at −20°C until use.

### Antibody functional analysis

BA and OPKA activity against the *S. sonnei* Moseley strain in serum or fecal supernatants were determined as previously described (19, 38). Briefly, for the BA, a bacteria suspension was mixed with heat-inactivated serially diluted test samples (serum or fecal supernatants) and baby rabbit complement (BRC, 12.5% final concentration) and incubated at 37°C for 2 h. For OPKA, bacteria was first opsonized with the heat-inactivated serially diluted test samples for 15 min at 37°C, followed by incubation with differentiated HL-60 cells (at a ratio of 200:1 of HL-60 cells to bacteria) and BRC (10% final concentration) at 37°C for 45 min. Luria-Bertani (LB) agar plates spotted with 10 µL of the reactions were grown overnight at 28°C. Titers, representing the reciprocal of the serum/fecal supernatant dilution that produced 50% bacterial killing, were calculated using Opsotiter (19). The provisional reference serum sample, Korean QC19, was run with each assay to normalize values (19). The lowest dilution tested was 1:4 (serum or fecal supernatant added into reaction neat); samples without bactericidal activity at this dilution were assigned a titer of 2.

### *S*. *sonnei* LPS-specific antibody isotype analysis

Fecal supernatants from 10 subjects vaccinated with 10^7^ CFU of WRSs2 (*n* = 5) or WRSs3 (*n* = 5), which exhibited the highest BA/OPKA titers at day 10, were selected for antibody isotype analysis. *S. sonnei* LPS-specific IgA and IgG titers were measured by ELISA. Briefly, Immulon 2HB plates 96-well plates (Thermo Fisher Scientific) were coated with *S. sonnei* LPS (Walter Reed Army Institute of Research) at 2.5 µg/mL in 100 µL carbonate buffer, pH 9.6, and incubated overnight at 4°C. After blocking for 1 hour at room temperature with 200 µL of 1× PBS containing 0.05% Tween-20 and 10% (wt/vol) non-fat dry milk, fecal supernatants serially diluted twofold starting at a 1:50 dilution were added and incubated at 37°C with shaking at 200 rpm for 90 min. Antibody detection was done using 100 µL of biotinylated antibody specific for human IgA or IgG (Thermo Fisher Scientific) for 1 hour at 37°C with shaking at 200 rpm, followed by a 1-hour incubation with streptavidin HRP (MilliporeSigma). Tetramethylbenzidine (KPL, Gaithersburg, MD) was added as substrate for 15 min in the dark with shaking, and the reaction was stopped by adding 100 µL of 1M phosphoric acid (MilliporeSigma). Endpoint titers were calculated as the inverse serum dilution that resulted in an absorbance value at 450 nm of 0.2 above background and were reported as the antibody titer.

### Fecal antibody purification

IgA and IgG were purified from each of the 10 fecal supernatants mentioned above using Agarose beads-peptide M (Invivogen, San Diego, CA) and melon gel (Thermo Fisher Scientific), respectively, using spin columns and following manufacturer’s instructions. The enrichment in each LPS-reactive antibody isotype and absence of the depleted isotype were confirmed by ELISA (described above).

### Statistical analyses

Differences in functional antibody levels in serum and fecal samples were evaluated using linear mixed effects models after log transformation of individual endpoint titers, taking into account the correlation among the multiple measurements from the same subject. Scheffe method was used to adjust for multiple comparisons and *P*-value <0.05 was considered statistically significant. A positive functional antibody response post-vaccination or seroconversion was defined as a greater than fourfold increase over the baseline titer. The overall percentage of SBA and OPKA seroconversion post-vaccination vs baseline and among the vaccine recipients and control group was compared by using Fisher’s exact test and analyzing kappa values. Associations between functional antibody titers were assessed using the Spearman rank correlation coefficient. Comparisons between shedding data and functional antibody responses were performed on log-transformed data using the Spearman rank correlation coefficient. Comparisons of functional antibody titers between paired antibody preparations from the same individual were performed using Wilcoxon matched-pairs signed rank test after log transformation of individual endpoint titers. All statistical comparisons were performed using STATA/SE version 17 (College Station, TX) and GraphPad Prism 9 (San Diego, CA).

## SUPPLEMENTAL MATERIAL

Tables S1 to S3 msphere.00419-23-s0001.docx Comparisons between post-vaccination titers and control or baseline titers.

ASM does not own the copyrights to Supplemental Material that may be linked to, or accessed through, an article. The authors have granted ASM a non-exclusive, world-wide license to publish the Supplemental Material files. Please contact the corresponding author directly for reuse.

## References

[1] Baker S, The HC. 2018. Recent insights into Shigella. Curr Opin Infect Dis 31:449–454. doi:10.1097/QCO.0000000000000475 30048255 PMC6143181

[2] Kotloff KL, Nataro JP, Blackwelder WC, Nasrin D, Farag TH, Panchalingam S, Wu Y, Sow SO, Sur D, Breiman RF, et al.. 2013. Burden and aetiology of diarrhoeal disease in infants and young children in developing countries (the global enteric multicenter study, GEMS): a prospective, case-control study. Lancet 382:209–222. doi:10.1016/S0140-6736(13)60844-2 23680352

[3] Nasrin D, Blackwelder WC, Sommerfelt H, Wu Y, Farag TH, Panchalingam S, Biswas K, Saha D, Jahangir Hossain M, Sow SO, et al.. 2021. Pathogens associated with linear growth faltering in children with diarrhea and impact of antibiotic treatment: the global enteric multicenter study. J Infect Dis 224:S848–S855. doi:10.1093/infdis/jiab434 34528677 PMC8958895

[4] Guerrant RL, DeBoer MD, Moore SR, Scharf RJ, Lima AAM. 2013. The impoverished gut--a triple burden of diarrhoea, stunting and chronic disease. Nat Rev Gastroenterol Hepatol 10:220–229. doi:10.1038/nrgastro.2012.239 23229327 PMC3617052

[5] Baker KS, Dallman TJ, Ashton PM, Day M, Hughes G, Crook PD, Gilbart VL, Zittermann S, Allen VG, Howden BP, et al.. 2015. Intercontinental dissemination of azithromycin-resistant shigellosis through sexual transmission: a cross-sectional study. Lancet Infect Dis 15:913–921. doi:10.1016/S1473-3099(15)00002-X 25936611

[6] Chung The H, Rabaa MA, Pham Thanh D, De Lappe N, Cormican M, Valcanis M, Howden BP, Wangchuk S, Bodhidatta L, Mason CJ, Nguyen Thi Nguyen T, Vu Thuy D, Thompson CN, Phu Huong Lan N, Voong Vinh P, Ha Thanh T, Turner P, Sar P, Thwaites G, Thomson NR, Holt KE, Baker S. 2016. South Asia as a reservoir for the global spread of ciprofloxacin-resistant Shigella sonnei: a cross-sectional study. PLoS Med 13:e1002055. doi:10.1371/journal.pmed.1002055 27483136 PMC4970813

[7] Kahsay AG, Muthupandian S. 2016. A review on sero diversity and antimicrobial resistance patterns of Shigella species in Africa, Asia and South America, 2001-2014. BMC Res Notes 9:422. doi:10.1186/s13104-016-2236-7 27576729 PMC5004314

[8] CDC. 2023. National antimicrobial resistance monitoring system (NARMS) now: human data. Available from: https://wwwn.cdc.gov/narmsnow

[9] Kotloff KL, Riddle MS, Platts-Mills JA, Pavlinac P, Zaidi AKM. 2018. Shigellosis. Lancet 391:801–812. doi:10.1016/S0140-6736(17)33296-8 29254859

[10] Ud-Din A, Wahid SUH, Latif HA, Shahnaij M, Akter M, Azmi IJ, Hasan TN, Ahmed D, Hossain MA, Faruque ASG, Faruque SM, Talukder KA. 2013. Changing trends in the prevalence of Shigella species: emergence of multi-drug resistant Shigella sonnei biotype G in Bangladesh. PLoS One 8:e82601. doi:10.1371/journal.pone.0082601 24367527 PMC3867351

[11] Thompson CN, Duy PT, Baker S. 2015. The rising dominance of Shigella sonnei: an intercontinental shift in the etiology of bacillary dysentery. PLoS Negl Trop Dis 9:e0003708. doi:10.1371/journal.pntd.0003708 26068698 PMC4466244

[12] Kozyreva VK, Jospin G, Greninger AL, Watt JP, Eisen JA, Chaturvedi V. 2016. Recent outbreaks of shigellosis in California caused by two distinct populations of Shigella sonnei with either increased virulence or fluoroquinolone resistance. mSphere 1:e00344-16. doi:10.1128/mSphere.00344-16 28028547 PMC5177732

[13] Raqib R, Qadri F, SarkEr P, Mia SMS, Sansonnetti PJ, Albert MJ, Andersson J. 2002. Delayed and reduced adaptive humoral immune responses in children with shigellosis compared with in adults. Scand J Immunol 55:414–423. doi:10.1046/j.1365-3083.2002.01079.x 11967124

[14] Van de Verg LL, Herrington DA, Boslego J, Lindberg AA, Levine MM. 1992. Age-specific prevalence of serum antibodies to the invasion plasmid and lipopolysaccharide antigens of Shigella species in chilean and North American populations. J Infect Dis 166:158–161. doi:10.1093/infdis/166.1.158 1607690

[15] Oberhelman RA, Kopecko DJ, Salazar-Lindo E, Gotuzzo E, Buysse JM, Venkatesan MM, Yi A, Fernandez-Prada C, Guzman M, León-Barúa R. 1991. Prospective study of systemic and mucosal immune responses in dysenteric patients to specific Shigella invasion plasmid antigens and lipopolysaccharides. Infect Immun 59:2341–2350. doi:10.1128/iai.59.7.2341-2350.1991 2050402 PMC258016

[16] Cohen D, Meron-Sudai S, Bialik A, Asato V, Goren S, Ariel-Cohen O, Reizis A, Hochberg A, Ashkenazi S. 2019. Serum IgG antibodies to Shigella lipopolysaccharide antigens - a correlate of protection against shigellosis. Hum Vaccin Immunother 15:1401–1408. doi:10.1080/21645515.2019.1606971 31070988 PMC6663123

[17] Cohen D, Ashkenazi S, Schneerson R, Farzam N, Bialik A, Meron-Sudai S, Asato V, Goren S, Baran TZ, Muhsen K, Gilbert PB, MacLennan CA. 2023. Threshold protective levels of serum IgG to Shigella lipopolysaccharide: re-analysis of Shigella vaccine trials data. Clin Microbiol Infect 29:366–371. doi:10.1016/j.cmi.2022.10.011 36243351 PMC9993342

[18] Necchi F, Saul A, Rondini S. 2017. Development of a high-throughput method to evaluate serum bactericidal activity using bacterial ATP measurement as survival readout. PLoS One 12:e0172163. doi:10.1371/journal.pone.0172163 28192483 PMC5305226

[19] Nahm MH, Yu J, Weerts HP, Wenzel H, Tamilselvi CS, Chandrasekaran L, Pasetti MF, Mani S, Kaminski RW. 2018. Development, Interlaboratory evaluations, and application of a simple, high-throughput Shigella serum bactericidal assay. mSphere 3:e00146-18. doi:10.1128/mSphere.00146-18 29898979 PMC6001606

[20] Ndungo E, Pasetti MF. 2020. Functional antibodies as immunological endpoints to evaluate protective immunity against Shigella. Hum Vaccin Immunother 16:197–205. doi:10.1080/21645515.2019.1640427 31287754 PMC7670857

[21] Boero E, Vezzani G, Micoli F, Pizza M, Rossi O. 2023. Functional assays to evaluate antibody-mediated responses against Shigella: a review. Front Cell Infect Microbiol 13:1171213. doi:10.3389/fcimb.2023.1171213 37260708 PMC10227456

[22] Sayem MA, Ahmad SM, Rekha RS, Sarker P, Agerberth B, Talukder KA, Raqib R. 2011. Differential host immune responses to epidemic and endemic strains of Shigella dysenteriae type I. J Health Popul Nutr 29:429–437. doi:10.3329/jhpn.v29i5.8896 22106748 PMC3225104

[23] Rahman MJ, Sarker P, Roy SK, Ahmad SM, Chisti J, Azim T, Mathan M, Sack D, Andersson J, Raqib R. 2005. Effects of zinc supplementation as adjunct therapy on the systemic immune responses in shigellosis. Am J Clin Nutr 81:495–502. doi:10.1093/ajcn.81.2.495 15699240

[24] Shimanovich AA, Buskirk AD, Heine SJ, Blackwelder WC, Wahid R, Kotloff KL, Pasetti MF. 2017. Functional and antigen-specific serum antibody levels as correlates of protection against shigellosis in a controlled human challenge study. Clin Vaccine Immunol 24:e00412-16. doi:10.1128/CVI.00412-16 27927680 PMC5299116

[25] Clarkson KA, Frenck RW, Dickey M, Suvarnapunya AE, Chandrasekaran L, Weerts HP, Heaney CD, McNeal M, Detizio K, Parker S, Hoeper A, Bourgeois AL, Porter CK, Venkatesan MM, Kaminski RW. 2020. Immune response characterization after controlled infection with lyophilized Shigella sonnei 53G. mSphere 5:e00988-19. doi:10.1128/mSphere.00988-19 32968012 PMC7568644

[26] Riddle MS, Kaminski RW, Di Paolo C, Porter CK, Gutierrez RL, Clarkson KA, Weerts HE, Duplessis C, Castellano A, Alaimo C, Paolino K, Gormley R, Gambillara Fonck V. 2016. Safety and immunogenicity of a candidate bioconjugate vaccine against Shigella flexneri 2a administered to healthy adults: a single-blind, randomized phase I study. Clin Vaccine Immunol 23:908–917. doi:10.1128/CVI.00224-16 27581434 PMC5139601

[27] Cohen D, Atsmon J, Artaud C, Meron-Sudai S, Gougeon M-L, Bialik A, Goren S, Asato V, Ariel-Cohen O, Reizis A, Dorman A, Hoitink CWG, Westdijk J, Ashkenazi S, Sansonetti P, Mulard LA, Phalipon A. 2021. Safety and immunogenicity of a synthetic carbohydrate conjugate vaccine against Shigella flexneri 2a in healthy adult volunteers: a phase 1, dose-escalating, single-blind, randomised, placebo-controlled study. Lancet Infect Dis 21:546–558. doi:10.1016/S1473-3099(20)30488-6 33186516

[28] Micoli F, Rossi O, Conti V, Launay O, Sciré AS, Aruta MG, Nakakana UN, Marchetti E, Rappuoli R, Saul A, Martin LB, Necchi F, Podda A. 2021. Antibodies elicited by the Shigella sonnei GMMA vaccine in adults trigger complement-mediated serum bactericidal activity: results from a phase 1 dose escalation trial followed by a booster extension. Front Immunol 12:671325. doi:10.3389/fimmu.2021.671325 34017343 PMC8129577

[29] Sarker P, Mily A, Ara A, Haque F, Maier N, Wierzba TF, Walker RI, Venkatesan MM, Raqib R. 2021. Functional antibodies and innate immune responses to WRSs1, a live oral Shigella sonnei vaccine candidate, in Bangladeshi adults and children. J Infect Dis 224:S829–S839. doi:10.1093/infdis/jiab395 34374425 PMC8687094

[30] Kapulu MC, Nakakana U, Sciré AS, Sarakinou E, Conti V, Rossi O, Acquaviva A, Necchi F, Obiero CW, Martin LB, Bejon P, Njuguna P, Micoli F, Podda A. 2022. Complement-mediated serum bactericidal activity of antibodies elicited by the Shigella sonnei GMMA vaccine in adults from a shigellosis-endemic country: exploratory analysis of a phase 2a randomized study. Front Immunol 13:971866. doi:10.3389/fimmu.2022.971866 36203568 PMC9531247

[31] Clarkson KA, Talaat KR, Alaimo C, Martin P, Bourgeois AL, Dreyer A, Porter CK, Chakraborty S, Brubaker J, Elwood D, Frölich R, DeNearing B, Weerts HP, Feijoo B, Halpern J, Sack D, Riddle MS, Fonck VG, Kaminski RW. 2021. Immune response characterization in a human challenge study with a Shigella flexneri 2a bioconjugate vaccine. EBioMedicine 66:103308. doi:10.1016/j.ebiom.2021.103308 33813141 PMC8047506

[32] Frenck RW, Conti V, Ferruzzi P, Ndiaye AGW, Parker S, McNeal MM, Dickey M, Granada JP, Cilio GL, De Ryck I, Necchi F, Suvarnapunya AE, Rossi O, Acquaviva A, Chandrasekaran L, Clarkson KA, Auerbach J, Marchetti E, Kaminski RW, Micoli F, Rappuoli R, Saul A, Martin LB, Podda A. 2021. Efficacy, safety, and immunogenicity of the Shigella sonnei 1790Gahb GMMA candidate vaccine: results from a phase 2b randomized, placebo-controlled challenge study in adults. EClinicalMedicine 39:101076. doi:10.1016/j.eclinm.2021.101076 34430837 PMC8367798

[33] Frenck RW, Baqar S, Alexander W, Dickey M, McNeal M, El-Khorazaty J, Baughman H, Hoeper A, Barnoy S, Suvarnapunya AE, Kaminski RW, Venkatesan MM. 2018. A phase I trial to evaluate the safety and immunogenicity of WRSs2 and WRSs3; two live oral candidate vaccines against Shigella sonnei. Vaccine 36:4880–4889. doi:10.1016/j.vaccine.2018.06.063 30037478 PMC10559265

[34] Hartman AB, Venkatesan MM. 1998. Construction of a stable attenuated Shigella sonnei deltavirg vaccine strain, WRSs1, and protective efficacy and immunogenicity in the guinea pig keratoconjunctivitis model. Infect Immun 66:4572–4576. doi:10.1128/IAI.66.9.4572-4576.1998 9712824 PMC108562

[35] Barnoy S, Jeong KI, Helm RF, Suvarnapunya AE, Ranallo RT, Tzipori S, Venkatesan MM. 2010. Characterization of WRSs2 and WRSs3, new second-generation virG(icsA)-based Shigella sonnei vaccine candidates with the potential for reduced reactogenicity. Vaccine 28:1642–1654. doi:10.1016/j.vaccine.2009.11.001 19932216 PMC2999844

[36] Barnoy S, Baqar S, Kaminski RW, Collins T, Nemelka K, Hale TL, Ranallo RT, Venkatesan MM. 2011. Shigella sonnei vaccine candidates WRSs2 and WRSs3 are as immunogenic as WRSs1, a clinically tested vaccine candidate, in a primate model of infection. Vaccine 29:6371–6378. doi:10.1016/j.vaccine.2011.04.115 21596086

[37] Venkatesan MM, Ballou C, Barnoy S, McNeal M, El-Khorazaty J, Frenck R, Baqar S. 2021. Antibody in lymphocyte supernatant (ALS) responses after oral vaccination with live Shigella sonnei vaccine candidates WRSs2 and WRSs3 and correlation with serum antibodies, ASCS, fecal IgA and shedding. PLoS One 16:e0259361. doi:10.1371/journal.pone.0259361 34793505 PMC8601580

[38] Ndungo E, Andronescu LR, Buchwald AG, Lemme-Dumit JM, Mawindo P, Kapoor N, Fairman J, Laufer MK, Pasetti MF. 2021. Repertoire of naturally acquired maternal antibodies transferred to infants for protection against shigellosis. Front Immunol 12:725129. doi:10.3389/fimmu.2021.725129 34721387 PMC8554191

[39] MacLennan CA, Grow S, Ma L, Steele AD. 2022. The Shigella vaccines pipeline. Vaccines 10:1376. doi:10.3390/vaccines10091376 36146457 PMC9504713

[40] Chang HS, Sack DA. 2001. Development of a novel in vitro assay (ALS assay) for evaluation of vaccine-induced antibody secretion from circulating mucosal lymphocytes. Clin Diagn Lab Immunol 8:482–488. doi:10.1128/CDLI.8.3.482-488.2001 11329444 PMC96087

[41] Chakraborty S, Harro C, DeNearing B, Bream J, Bauers N, Dally L, Flores J, Van de Verg L, Sack DA, Walker R, Staats HF. 2016. Evaluation of the safety, tolerability, and immunogenicity of an oral, inactivated whole-cell Shigella flexneri 2a vaccine in healthy adult subjects. Clin Vaccine Immunol 23:315–325. doi:10.1128/CVI.00608-15 26865592 PMC4820506

[42] Chen WH, Cohen MB, Kirkpatrick BD, Brady RC, Galloway D, Gurwith M, Hall RH, Kessler RA, Lock M, Haney D, Lyon CE, Pasetti MF, Simon JK, Szabo F, Tennant S, Levine MM. 2016. Single-dose live oral cholera vaccine CVD 103-HgR protects against human experimental infection with Vibrio cholerae O1 El Tor. Clin Infect Dis 62:1329–1335. doi:10.1093/cid/ciw145 27001804 PMC4872293

[43] Holmgren J, Svennerholm AM. 1977. Mechanisms of disease and immunity in cholera: a review. J Infect Dis 136 Suppl:S105–12. doi:10.1093/infdis/136.supplement.s105 197173

[44] Roos A, Bouwman LH, van Gijlswijk-Janssen DJ, Faber-Krol MC, Stahl GL, Daha MR. 2001. Human IgA activates the complement system via the mannan-binding lectin pathway. J Immunol 167:2861–2868. doi:10.4049/jimmunol.167.5.2861 11509633

[45] Itami H, Hara S, Samejima K, Tsushima H, Morimoto K, Okamoto K, Kosugi T, Kawano T, Fujiki K, Kitada H, Hatakeyama K, Tsuruya K, Ohbayashi C. 2020. Complement activation is associated with crescent formation in IgA nephropathy. Virchows Arch 477:565–572. doi:10.1007/s00428-020-02800-0 32300880

[46] Medjeral-Thomas NR, Cook HT, Pickering MC. 2021. Complement activation in IgA nephropathy. Semin Immunopathol 43:679–690. doi:10.1007/s00281-021-00882-9 34379175 PMC8551128

[47] Boullier S, Tanguy M, Kadaoui KA, Caubet C, Sansonetti P, Corthésy B, Phalipon A. 2009. Secretory IgA-mediated neutralization of Shigella flexneri prevents intestinal tissue destruction by down-regulating inflammatory circuits. J Immunol 183:5879–5885. doi:10.4049/jimmunol.0901838 19828639

[48] Mathias A, Longet S, Corthésy B. 2013. Agglutinating secretory IgA preserves intestinal epithelial cell integrity during apical infection by Shigella flexneri. Infect Immun 81:3027–3034. doi:10.1128/IAI.00303-13 23753631 PMC3719585

[49] Brandtzaeg P. 2009. Mucosal immunity: induction, dissemination, and effector functions. Scand J Immunol 70:505–515. doi:10.1111/j.1365-3083.2009.02319.x 19906191

[50] Tacket CO, Binion SB, Bostwick E, Losonsky G, Roy MJ, Edelman R. 1992. Efficacy of bovine milk immunoglobulin concentrate in preventing illness after Shigella flexneri challenge. AmJTropMedHyg 47:276–283. doi:10.4269/ajtmh.1992.47.276 1524140

[51] Hurley WL, Theil PK. 2011. Perspectives on immunoglobulins in colostrum and milk. Nutrients 3:442–474. doi:10.3390/nu3040442 22254105 PMC3257684

[52] Kunz N, Kemper C. 2021. Complement has brains-do intracellular complement and immunometabolism cooperate in tissue homeostasis and behavior? Front Immunol 12:629986. doi:10.3389/fimmu.2021.629986 33717157 PMC7946832

[53] Noel G, In JG, Lemme-Dumit JM, DeVine LR, Cole RN, Guerrerio AL, Campbell JD, Kovbasnjuk O, Pasetti MF. 2021. Human breast milk enhances intestinal mucosal barrier function and innate immunity in a healthy pediatric human enteroid model. Front Cell Dev Biol 9:685171. doi:10.3389/fcell.2021.685171 34327199 PMC8313895

[54] Kopp ZA, Jain U, Van Limbergen J, Stadnyk AW. 2015. Do antimicrobial peptides and complement collaborate in the intestinal mucosa? Front Immunol 6:17. doi:10.3389/fimmu.2015.00017 25688244 PMC4311685

[55] Wirthmueller U, Dewald B, Thelen M, Schäfer MK, Stover C, Whaley K, North J, Eggleton P, Reid KB, Schwaeble WJ. 1997. Properdin, a positive regulator of complement activation, is released from secondary granules of stimulated peripheral blood neutrophils. J Immunol 158:4444–4451. 9127010

[56] Kemper C, Atkinson JP, Hourcade DE. 2010. Properdin: emerging roles of a pattern-recognition molecule. Annu Rev Immunol 28:131–155. doi:10.1146/annurev-immunol-030409-101250 19947883

[57] Wu M, Zheng W, Song X, Bao B, Wang Y, Ramanan D, Yang D, Liu R, Macbeth JC, Do EA, Andrade WA, Yang T, Cho H-S, Gazzaniga FS, Ilves M, Coronado D, Thompson C, Hang S, Chiu IM, Moffitt JR, Hsiao A, Mekalanos JJ, Benoist C, Kasper DL. 2023. Microbiome induced complement synthesized in the gut protects against enteric infections. bioRxiv:2023.02.02.523770. doi:10.1101/2023.02.02.523770

[58] Kotloff KL, Taylor DN, Sztein MB, Wasserman SS, Losonsky GA, Nataro JP, Venkatesan M, Hartman A, Picking WD, Katz DE, Campbell JD, Levine MM, Hale TL. 2002. Phase I evaluation of delta virG Shigella sonnei live, attenuated, oral vaccine strain WRSs1 in healthy adults. Infect Immun 70:2016–2021. doi:10.1128/IAI.70.4.2016-2021.2002 11895966 PMC127867

[59] Pitisuttithum P, Islam D, Chamnanchanunt S, Ruamsap N, Khantapura P, Kaewkungwal J, Kittitrakul C, Luvira V, Dhitavat J, Venkatesan MM, Mason CJ, Bodhidatta L, Pasetti MF. 2016. Clinical trial of an oral live Shigella sonnei vaccine candidate, WRSs1, in Thai adults. Clin Vaccine Immunol 23:564–575. doi:10.1128/CVI.00665-15 27146000 PMC4933782

[60] Lemme-Dumit JM, Doucet M, Zachos NC, Pasetti MF. 2022. Epithelial and neutrophil interactions and coordinated response to Shigella in a human intestinal enteroid-neutrophil coculture model. mBio 13:e0094422. doi:10.1128/mbio.00944-22 35652591 PMC9239269

