# Supplementary material for: Oral immunization with Shigella sonnei WRSs2 and WRSs3 vaccine strains elicits systemic and mucosal antibodies with functional anti-microbial activity
Source: mSphere. 2023 Dec 22;9(1):e00419-23. doi: 10.1128/msphere.00419-23 (PMC10826362; doi:10.1128/msphere.00419-23)
Supplement: Tables S1 to S3 — Comparisons between post-vaccination titers and control or baseline titers. [file msphere.00419-23-s0001.docx]

| Supplementary Table S1. Comparisons between post-vaccination systemic SBA and OPKA titers and placebo (control) titers or baseline titers. | | | | | | | | | | |
| --- | --- | --- | --- | --- | --- | --- | --- | --- | --- | --- |
|  |  |  | **WRSs2** | | | | **WRSs3** | | | |
|  |  |  | SBA | | OPKA | | SBA | | OPKA | |
|  | Comparator | Placebo  (n= 8) | 10^6^ CFU  (n= 7) | 10^7^ CFU  (n= 7) | 10^6^ CFU  (n= 7) | 10^7^ CFU  (n= 7) | 10^6^ CFU  (n= 8) | 10^7^ CFU  (n= 7) | 10^6^ CFU  (n= 8) | 10^7^ CFU  (n= 7) |
| Day 7 | vs Control | Reference | 0.0389* | 0.0019** | 0.4834 | 0.0000*** | 0.2197 | 0.0851 | 0.1458 | 0.1208 |
|  | vs Baseline |  | 0.0000*** | 0.0049** | 0.7178 | 0.0005*** | 0.2257 | 0.0000*** | 0.1181 | 0.0266* |
| Day 14 | vs Control | Reference | 0.0000*** | 0.0000*** | 0.0099** | 0.0000*** | 0.0001*** | 0.0000*** | 0.0000*** | 0.0000*** |
|  | vs Baseline |  | 0.0000*** | 0.0000*** | 0.0015** | 0.0000*** | 0.0000*** | 0.0000*** | 0.0000*** | 0.0000*** |
| Day 28 | vs Control | Reference | 0.0001*** | 0.0000*** | 0.0315* | 0.0000*** | 0.0031* | 0.0000*** | 0.0160* | 0.0000*** |
|  | vs Baseline |  | 0.0000*** | 0.0000*** | 0.0138* | 0.0000*** | 0.0006*** | 0.0000*** | 0.0020** | 0.0000*** |
| Day 56 | vs Control | Reference | 0.0074** | 0.0150* | 0.1124 | 0.0001*** | 0.1765 | 0.0949 | 0.3919 | 0.3334 |
|  | vs Baseline |  | 0.0000*** | 0.0545 | 0.1391 | 0.0049** | 0.1181 | 0.0000*** | 0.4582 | 0.1126 |
| Table shows *P* values from comparisons at different time points using linear mixed effect model after log-transformation of individual endpoint titers. **P* < 0.05, ** *P* < 0.01, ****P* < 0.001. | | | | | | | | | | |

| **Supplementary Table S2. Comparisons between post-vaccination mucosal BA and OPKA titers and placebo (control) titers or baseline titers.** | | | | | | | | | | |
| --- | --- | --- | --- | --- | --- | --- | --- | --- | --- | --- |
|  |  |  | **WRSs2** | | | | **WRSs3** | | | |
|  |  |  | Fecal BA | | Fecal OPKA | | Fecal BA | | Fecal OPKA | |
|  | Comparator | Placebo  (n= 8) | 10^6^ CFU  (n= 7) | 10^7^ CFU (n= 7) | 10^6^ CFU (n= 7) | 10^7^ CFU (n= 7) | 10^6^ CFU (n= 8) | 10^7^ CFU  (n= 7) | 10^6^ CFU (n= 8) | 10^7^ CFU (n= 7) |
| Day 7 | vs Control | Reference | 0.9717 | 0.0002*** | 0.9855 | 0.0851 | 0.4663 | 0.1759 | 0.5597 | 0.1895 |
|  | vs Baseline |  | 0.9678 | 0.0000*** | 0.9839 | 0.0607 | 0.3968 | 0.1299 | 0.5043 | 0.1481 |
| Day 10 | vs Control | Reference | 0.0000*** | 0.0000*** | 0.0000*** | 0.0000*** | 0.0001*** | 0.0000*** | 0.0000*** | 0.0000*** |
|  | vs Baseline |  | 0.0000*** | 0.0000*** | 0.0000*** | 0.0000*** | 0.0000*** | 0.0000*** | 0.0000*** | 0.0000*** |
| Day 14 | vs Control | Reference | 0.0112* | 0.0996 | 0.0780 | 0.3148 | 0.3176 | 0.0065** | 0.6639 | 0.0273* |
|  | vs Baseline |  | 0.0013** | 0.0545 | 0.0498* | 0.2804 | 0.1600 | 0.0007*** | 0.6188 | 0.0140* |
| Day 28 | vs Control | Reference | 0.9702 | 0.8834 | 0.9848 | 0.9211 | 0.9825 | 0.5571 | 0.9911 | 0.9330 |
|  | vs Baseline |  | 0.9678 | 0.9805 | 0.9839 | 0.9180 | 0.9805 | 0.5327 | 0.9902 | 0.9298 |
| Table shows *P* values from comparisons at different time points using linear mixed effect model after log-transformation of individual endpoint titers. **P* < 0.05, ** *P* < 0.01, ****P* < 0.001. | | | | | | | | | | |

| **Supplementary Table S3. Association between functional antibody responses and shedding in WRSs2 and WRSs3 recipients.** | | | | | | | |
| --- | --- | --- | --- | --- | --- | --- | --- |
|  | Spearman rank correlation (95% CI) | | | | | | |
|  | **WRSs2 recipients (n = 14)** | | **WRSs3 recipients (n = 15)** | | **All individuals**  **(vaccinees and placebo, n = 37)** | | |
|  | Peak CFU/gm | Duration of shedding (days) | Peak CFU/gm | Duration of shedding (days) | | Peak CFU/gm | Duration of shedding (days) |
| **Peak titers:** |  |  |  |  | |  |  |
| SBA | 0.13 (-0.44, 0.63) | 0.20 (-0.39, 0.67) | 0.21 (-0.35, 0.66) | 0.13 (-0.43, 0.61) | | 0.49 (0.18, 0.70) | 0.47 (0.16, 0.69) |
| OPKA | -0.33 (-0.74, 0.26) | -0.22 (-0.68, 0.37) | 0.23 (-0.33, 0.67) | 0.14 (-0.42, 0.62) | | 0.20 (-0.14, 0.50) | 0.25 (-0.10, 0.53) |
| Fecal BA | -0.06 (-0.59, 0.50) | 0.22 (-0.36, 0.68) | -0.13 (-0.61, 0.43) | -0.07 (-0.57, 0.47) | | 0.31 (-0.02, 0.58) | 0.40 (0.08, 0.65) |
| Fecal OPKA | 0.02 (-0.53, 0.55) | 0.22 (-0.37, 0.68) | -0.12 (-0.61, 0.43) | -0.09 (-0.59, 0.46) | | 0.37 (0.04, 0.62) | 0.45 (0.13, 0.68) |
| **Peak fold rise:** |  |  |  |  | |  |  |
| SBA | 0.34 (-0.25, 0.74) | 0.33 (-0.26, 0.74) | 0.33 (-0.23, 0.73) | 0.35 (-0.22, 0.74) | | 0.61 (0.34, 0.78) | 0.61 (0.35, 0.79) |
| OPKA | 0.28 (-0.31, 0.71) | 0.18 (-0.41, 0.66) | 0.27 (-0.30, 0.70) | 0.27 (-0.29, 0.70) | | 0.49 (0.19, 0.71) | 0.49 (0.19, 0.71) |
| Fecal BA | -0.04 (-0.57, 0.51) | 0.23 (-0.36, 0.69) | -0.02 (-0.54, 0.51) | -0.06 (-0.57, 0.48) | | 0.39 (0.06, 0.64) | 0.44 (0.13, 0.68) |
| Fecal OPKA | 0.02 (-0.53, 0.55) | 0.22 (-0.37, 0.68) | -0.02 (-0.54, 0.51) | -0.06 (-0.57, 0.48) | | 0.42 (0.10, 0.66) | 0.48 (0.18-0.70) |
